# Experiences of frail older cardiac patients with a nurse-coordinated transitional care intervention - a qualitative study

**DOI:** 10.1186/s12913-021-06719-3

**Published:** 2021-08-10

**Authors:** Patricia Jepma, Corine H. M. Latour, Iris H. J. ten Barge, Lotte Verweij, Ron J. G. Peters, Wilma J. M. Scholte op Reimer, Bianca M. Buurman

**Affiliations:** 1 Department of Cardiology, Amsterdam UMC, University of Amsterdam, Amsterdam, The Netherlands; 2 Center of Expertise Urban Vitality, Faculty of Health, Amsterdam University of Applied Sciences, Amsterdam, The Netherlands; 3 Nursing Sciences, Program of Clinical Health Sciences, University Medical Center Utrecht, Utrecht, The Netherlands; 4 HU University of Applied Sciences Utrecht, Research Group Chronic Diseases, Utrecht, The Netherlands; 5 Department of Internal Medicine, Section of Geriatric Medicine, Amsterdam UMC, University of Amsterdam, Amsterdam, The Netherlands

**Keywords:** Cardiac rehabilitation, Cardiology, Case management, Disease management, Frailty, Nurses, Physical therapists, Qualitative research, Transitional care

## Abstract

**Background:**

Older cardiac patients are at high risk of readmission and mortality. Transitional care interventions (TCIs) might contribute to the prevention of adverse outcomes. The Cardiac Care Bridge program was a randomized nurse-coordinated TCI combining case management, disease management and home-based rehabilitation for hospitalized frail older cardiac patients. This qualitative study explored the experiences of patients’ participating in this study, as part of a larger process evaluation as this might support interpretation of the neutral study outcomes. In addition, understanding these experiences could contribute to the design and application of future transitional care interventions for frail older cardiac patients.

**Methods:**

A generic qualitative approach was used. Semi-structured interviews were performed with 16 patients ≥70 years who participated in the intervention group. Participants were selected by gender, diagnosis, living arrangement and hospital of inclusion. Data were analysed using thematic analysis. In addition, quantitative data about intervention delivery were analysed.

**Results:**

Three themes emerged from the data: 1) appreciation of care continuity; 2) varying experiences with recovery and, 3) the influence of an existing care network. Participants felt supported by the transitional care intervention as they experienced post-discharge support and continuity of care. The perceived contribution of the program in participants’ recovery varied. Some participants reported physical improvements while others felt impeded by comorbidities or frailty. The home visits by the community nurse were appreciated, although some participants did not recognize the added value. Participants with an existing healthcare provider network preferred to consult these providers instead of the providers who were involved in the transitional care intervention.

**Conclusion:**

Our results contribute to an explanation of the neutral study of a nurse-coordinated transitional care intervention. For future purpose, it is important to identify which patients might benefit most from TCIs. Furthermore, the intensity and content of TCIs could be more personalized by tailoring interventions to older cardiac patients’ needs, considering their frailty, self-management skills and existing formal and informal caregiver networks.

**Supplementary Information:**

The online version contains supplementary material available at 10.1186/s12913-021-06719-3.

## Background

Older cardiac patients are at high risk of hospital readmission and mortality, especially in the first weeks after hospitalization for a cardiac event [1, 2]. The simultaneously presence of both cardiac and geriatric conditions increase these risks [3, 4], e.g. non-adherence in cognitively impaired heart failure patients or poor participation in cardiac rehabilitation (CR) because of disabling comorbidities and intensive centre-based programs [5, 6]. In addition, the risk of readmission and mortality is increased by inadequate care transitions [7].

Transitional care interventions (TCIs) aim to improve continuity of care in patients transitioning between care settings and are usually provided by a case management approach with a broad focus on patients’ needs [7]. These interventions have been proven to reduce hospital readmission and mortality in older and chronically ill patients [8, 9]. However, the results of transitional care interventions in cardiac patients show mixed results on these outcomes [10–12]. Besides case management, (older) cardiac patients also need disease-specific guidance post-discharge regarding symptom monitoring, medication and lifestyle-related adherence and cardiac rehabilitation (CR).

The Cardiac Care Bridge (CCB) program was a nurse-coordinated TCI combining case management, disease management and home-based CR for frail hospitalized cardiac patients ≥70 years. This TCI was a complex intervention as it included multiple interacting intervention components, stakeholders and organisational levels [13]. No statistically significant difference was found on the main composite outcome of readmission and mortality within 6 months after randomization [14]. Besides analysing trial outcomes, we performed a process evaluation to examine the mechanisms and contextual factors that influenced these outcomes. Trial participants are important stakeholders who do not passively receive, but actually interact with the intervention [15]. More knowledge on their perspectives regarding the intervention is valuable as their perspectives might contribute to an interpretation of the study outcomes. In addition, understanding these experiences could contribute to the design and application of future transitional care interventions for frail older cardiac patients.

## Methods

### Aim

The aim of this study was to explore the experiences of participants who received a nurse-coordinated TCI in order to support interpretation of the study outcomes from participants’ perspective.

### Design

We used a generic qualitative approach to understand participants’ experiences with a nurse-coordinated TCI [16]. This design was considered suitable as the research question did not fit any of the established methodologies (e.g. grounded theory, phenomenology and ethnography) [17]. The generic qualitative approach allowed us to use the strengths of these methodologies and the flexibility to gather a rich and in-depth description of participants’ experiences. COREQ-guidelines have been used for transparency reporting [18].

### Participants

Participants of this qualitative study were frail cardiac patients ≥70 years who were included in the intervention group of a TCI within the last 3 months [19]. Participants were purposively selected by gender, diagnosis, living arrangement (alone/together) and hospital of inclusion to ensure a maximum variation of experiences. They were invited by phone to participate in an interview after the intervention was completed. Recruitment stopped when no new codes and themes emerged from the data and the research question could be answered [20].

### The cardiac care bridge transitional care program

The CCB program was a Dutch multi-center randomized controlled trial (RCT) on nurse-coordinated, interdisciplinary transitional care in frail, older (≥70 years) hospitalized cardiac patients. In total, 306 patients were recruited in six hospitals. The primary outcome was a composite endpoint of unplanned hospital readmission and mortality within 6 months after randomization.

**Fig. 1:**
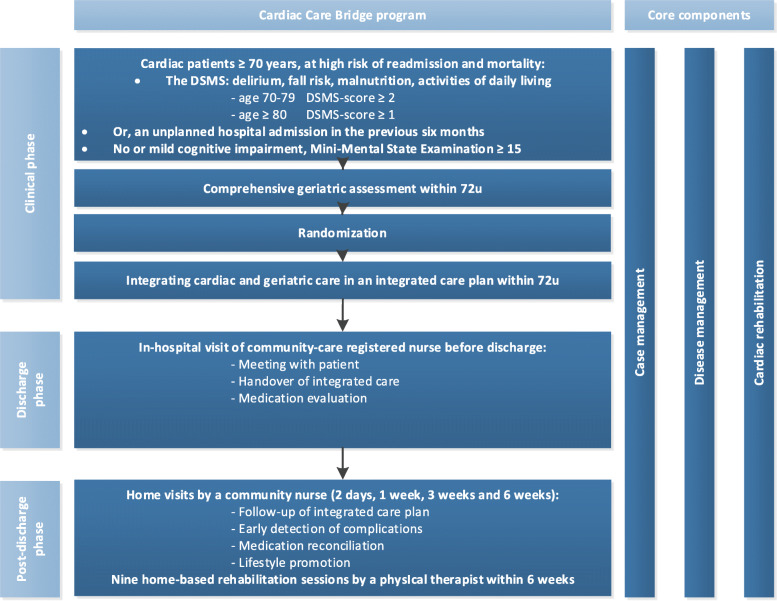
Overview of the Cardiac Care Bridge transitional care program

The CCB program was a complex intervention combining case management, disease management and CR in three phases; the clinical, discharge and post-discharge phase (Fig. 1). In the clinical phase, a comprehensive geriatric assessment (CGA) was performed to identify geriatric conditions and to develop an integrated care plan. This plan was leading in care provided by the involved healthcare providers during the three phases of the TCI. In the discharge phase, a community-care registered nurse (CN) visited the patient and clinical healthcare providers in the hospital for a personal handover of the integrated care plan and to prepare the discharge phase. In the post-discharge phase, the CN performed four home visits within the first 6 weeks. These home visits included among others medication reconciliation, early signalling of health deterioration or complications and an evaluation of the integrated care plan. On indication, an extra home visit was performed within the first 3 months post-discharge. In addition, a physical therapist (PT) performed nine home-based CR sessions at patients’ home. Details of this study have been published [19].

### Data collection

The interviews were conducted between December 2017 and June 2018 at participants’ home. They were interviewed alongside the ongoing trial, but only when the intervention in the post-clinical phase was finished. Some participants requested the presence of an informal caregiver which was allowed by the researchers. The interviews were performed by two researchers (PJ or IB) who followed additional training in qualitative research. Both have a Bachelor degree in nursing. PJ also has a Master degree in health sciences. She examined the integration of cardiac and geriatric care in older patients with heart disease. IB followed a nursing Master programme during the time of the interviews and worked as a quality nurse in an organization for nursing homes. Both researchers did not had prior relations with the included participants. A semi-structured interview guide (see Additional file 1) was developed based on the clinical, discharge and post-discharge phase of the intervention. Small adjustments have been made during the data collection process to ensure that all key elements of the intervention were fully questioned. The complete interviews were audio recorded and field notes were made during and after the interview. The interviews lasted between 25 and 70 min.

Data regarding participants sociodemographic and disease characteristics were collected for the RCT during hospitalization (Table 1). Furthermore, data about the intervention delivery was registered in medical hospital files and logbooks which were filled out by the participating healthcare providers during the intervention. A process evaluation of the intervention delivery is reported [24].

**Table 1:** Baseline characteristics of interviewed participants in the Cardiac Care Bridge program

Patient	Age	Sex	Residency status	Educational level	MMSE^**a**^	DSMS^**b**^	Hospitali-zation ≤ 6 months	Primary diagnosis	Charlson comorbidity index^**c**^
1	87	Female	With partner	Primary	21	1	No	Valve deficit	2
2	86	Female	Alone	Primary	29	1	No	Acute coronary syndrome	2
3	81	Male	With partner	Primary	27	1	No	Heart failure	4
4	85	Female	Alone	Secondary	24	2	No	Acute coronary syndrome	0
5	76	Male	With partner	Higher	27	3	Yes	Rhytm or conduction disorder	4
6	87	Male	Alone	Secondary	29	1	No	Heart failure	4
7	84	Female	Alone	Higher	28	3	No	Heart failure	2
8	82	Male	With partner	Higher	28	1	No	Rhytm or conduction disorder	6
9	89	Male	With child	Higher	27	1	No	Heart failure	4
10	82	Female	With partner	Primary	29	2	Yes	Heart failure	1
11	79	Male	With partner	Higher	29	2	No	Rhytm or conduction disorder	1
12	87	Female	Alone	Primary	29	1	Yes	Valve deficit	3
13	73	Male	Alone	Primary	24	1	Yes	Heart failure	5
14	86	Male	Alone	Secondary	27	4	No	Heart failure	2
15	84	Female	Alone	Primary	24	1	No	Heart failure	4
16	71	Female	With partner	Primary	24	3	Yes	Heart failure	5

^a^ Mini-Mental State Examination [21]: the score ranges between 0 and 30 points. In the CCB program, only patients with a MMSE ≥15 were included. A MMSE-score < 24 indicates cognitive impairment. ^b^ Dutch Safety Management System [22]: the score between 0 and 4 points, based on four domains of frailty (malnutrition, risk of impairments in daily functioning, risk on delirium and fall risk). A higher score on the DSMS indicates a higher risk of functional loss. ^c^ Charlson comorbidity index [23]: a weighted index to classify comorbid conditions based on their 1-year mortality prognosis

### Ethical consideration

The CCB study was approved by the Medical Research Ethics Committee of the AMC (Protocol ID: MEC2016_024) (Netherlands Trial Register number: NTR6316, 06/04,2017) and conforms with the principles outlined in the declaration of Helsinki [25]. Prior to the interview, participants received oral and written information about this qualitative study and written informed consent was obtained.

### Data analysis

Two researchers (PJ and IB) were involved in the data analysis. Data were analysed by the assumption that this TCI might have beneficial effects on readmission and mortality and that participants might therefore be positive about the intervention. To prevent potential bias, the qualitative data on participants’ perspectives were analysed before the study results on effectiveness of the TCI were known. However, the researchers were involved in the data collection on the intervention delivery in the large process evaluation [24] and therefore aware of a suboptimal intervention fidelity. This information was taken into account into the data analysis of participants’ experiences in order to understand the findings.

The six phases of thematic analysis according to Braun and Clarke [26] were used to analyse the data. All interviews were transcribed verbatim. PJ and IB familiarized themselves with the data by reading the transcripts (phase 1). The coding process started with open coding using the coding program MAXQDA 12. Per two coded transcripts, consensus about codes was reached before coding the next two transcripts (phases 2). During this process, PJ and IB discussed emerging themes (phase 3) which were reviewed repeatedly and discussed with the research team (phase 4). All codes were analysed and structured, which led to the final themes (phase 5). Corresponding quotes were selected, the research question was answered and findings were compared with literature (phase 6).

We also analysed quantitative data about the delivery of intervention key elements (e.g. in-hospital personal handover, home visits) in interviewed participants from medical hospital files and logbooks (Table 2). This additional information contributed to a complete view of the intervention delivery in interviewed participants and helped to put participants’ experiences in the context of delivered care.

**Table 2:** Intervention delivery in interviewed participants

Patient	Clinical phase			Discharge phase	Post-discharge phase						
	CGA	Geriatric consultation	Geriatric consultation indicated?^**a**^	Handover	Number of home visits CN^**b**^	First home visit CN within median of 3 days	Medication verification	Evaluation of care plan	Lifestyle discussed	Number of home visits PT^**c**^	Joint intake CN/PT
1	Yes	No	Yes	Face to face	4	No	Yes	No	Yes	4	Yes
2	Yes	No	Yes	Face to face	4	Yes	Yes	No	No	9	No
3	Yes	No	Yes	Face to face	4	Yes	Yes	No	Yes	9	No
4	Yes	No	Yes	Telephone	3	No	Yes	Yes	Yes	6	Yes
5	Yes	No	Yes	Face to face	5	No	Yes	Yes	Yes	7	No
6	Yes	No	Yes	Unknown	4	Yes	Yes	Yes	Yes	9	No
7	Yes	No	Yes	Telephone	4	Yes	Yes	Yes	Yes	9	No
8	Yes	No	Yes	Unknown	5	No	Yes	Yes	Yes	8	No
9	Yes	No	No	Face to face	4	Yes	Yes	No	Yes	9	Yes
10	Yes	No	No	Telephone	3	No	Yes	No	No	9	No
11	Yes	No	No	Face to face	4	Yes	Yes	Yes	Yes	1	NA
12	Yes	No	No	Face to face	4	Yes	Yes	Yes	No	0	NA
13	Yes	No	No	Face to face	4	Yes	Yes	Yes	Yes	4	No
14	Yes	No	No	Telephone	5	Yes	Yes	Yes	Yes	7	No
15	Yes	Yes	Yes	Face to face	5	Yes	Yes	No	Yes	7	No
16	Yes	No	Yes	Face to face	3	Yes	Yes	No	Yes	2	No

*Abbreviations*: *CGA* comprehensive geriatric assessment, *CN* community nurse, *NA* not applicable, *PT* physical therapist

^**a**^ Geriatric team consultation was indicated in case of ≥5 geriatric problems of which ≥1 problem had to be within the psychological domain. ^**b**^ Four home visits, according to the intervention protocol. An extra home visit was performed on indication, assessed by the CN. ^c^ Max. nine home-based rehabilitation session, according to the intervention protocol

## Results

Data saturation was reached after 16 interviews. Participants’ partner or a child participated in eight interviews. The mean age of included participants was 82.4 years (SD 5.3), 50% was female, 56.3% was admitted due to heart failure and 56.3% lived together with a partner (Table 1). Table 2 shows the intervention delivery in interviewed participants. In total, three themes were identified from the interviews: 1) appreciation of care continuity; 2) varying experiences with recovery and, 3) the influence of an existing care network.

### Theme 1: appreciation of care continuity

Participants experienced that healthcare providers during all three phases of care (clinical, discharge and post-discharge phase) looked after them. During the clinical phase, participants reported that they met many different healthcare providers and most participants were unable to distinguish usual hospital care from the care delivered in the TCI. Most experiences of participants were therefore about the discharge and post-discharge phase of this TCI. During the discharge phase, the CGA-based integrated care plan was discussed by the cardiac research nurse during a face-to-face handover with the community nurse, in the presence of the patient. Participants appreciated it to meet the community nurse before discharge to be prepared for who would visit them at home:

*‘She said this is the nursing service that comes to your home, ( … ). Well I think that is neat. ( … ) Look, you know who you are dealing with and not that umm there suddenly is one at the door and you think hey … This feels good.’ (*P13, male, 73 years)

In the post-discharge phase, participants reported that they were satisfied about the relationship with the healthcare providers because they felt that providers were experienced, adequately informed about their health and kept an extra eye on them post-discharge. As the following participant stated, this also led to more motivation to the home-based CR exercises:

*‘Yes, above all that, you get guidance and a helping hand to keep doing it [physical exercises]. Look, if you throw in at the deep end now and you have to do exercises, then it will either happen or not. But she [the physical therapist] was really adamant that "well you have to do it". Well then you simply just did. I was happy with it [with the TCI]. That gives you some certainty.’* (P5, male, 76 years)

Regarding the community nurse, participants experienced support in checking their health status by measurement of vital signs. They also felt supported in medication management. For example, one participant had specific goals about her medication adherence and the community nurse arranged a multi-dose drug dispenser for her:

*‘I say, I do need that [the community nurse] every now and then. There is a big stick behind the door (...) That it was said and now you should do that. And now you really have to make sure you take your pills on time.’* (P16, female, 71 years)

Participants had some difficulties to fully describe the care that was delivered by the community nurse. Additional information from the logbooks showed that the community nurse performed medication reconciliation in all participants. Furthermore, in 9/16 participants the integrated care plan was evaluated and in 13/16 participants lifestyle promotion was discussed (Table 2). Furthermore, in 3/15 participants a joint home visit of the community nurse and physical therapist was performed to coordinate care together.

### Theme 2: varying experiences with recovery

The majority of participants were satisfied about their recovery in the post-discharge phase. Participants reasoned that, as part of aging, recovery took time or understood that recovery was not fully feasible.

All participants received home visits of the community nurse post-discharge. The number of home visits by the community nurse ranged from three to five (mean = 4, SD 0.7) and by the physical therapist from zero to nine (median = 7, [IQR: 4–9]) (Table 2). Many participants indicated that the number of home visits by the community nurse and physical therapist were sufficient, and more care would not have contributed to their recovery.

Regarding the home visits by the community nurse, some participants reported interventions by the community nurse, but not recognized the importance in relation to the prevention of further complications.

Therefore, it was difficult for participants to recall if and how the community nurse had contributed to their recovery:

*‘Yes, I do not really know [whether the community nurses contributed]. No, as I am now, I actually feel good physically, except for that wound and my feet. But they cannot do anything about that anymore. I like it when she visits. But whether it contributes [home visits of the community nurse] that I doubt.’* (P9, male, 89 years)

In general, participants considered the home-based CR as an opportunity to work on their daily functioning. Participants with personal goals were motivated to achieve progress in their recovery:

*‘Because I also say last time, “I have set a goal, I want to be able to walk for an hour and I want to be able to cycle a bit again”, and then he says [physical therapist] “well for the last couple of times we will try to cycle together”.’* (P10, female, 82 years).

Participants experienced progress in their recovery mainly in improved muscle strength and condition:

*‘Look, I can do all those exercises, and, in the beginning, you were uhm well then you really had to catch up. But now I just recover in a minute, two minutes and then it is back to normal.*

*So, then you see, you feel that you are building up something and that is important.’* (P5, male, 76 years)

However, most participants were severe frail or were limited duo to comorbidities. One participant therefore ended the home-based CR prematurely. In other participants, the experienced symptoms (e.g. dyspnoea, tiredness, joint problems) impeded them during the physical exercises:

*‘Yes, and that did not work [the exercises], it is too tiring for me, for my legs. ( … ). For a young guy, the suggested exercises were good, you have to be of the right age. But my whole body will be gone in a minute*.’ (P3, male, 81 years)

In addition to the rehabilitation sessions, most participants received exercises to practice on a daily basis without the presence of the physical therapist. Participants indicated that they often forgot to practise or found it hard to fit these exercises in their daily routine:

*‘You do not get to it when you are alone. Then we have, when I remember that I have to do it [exercises of the physical therapist], I have something again, then I had to turn off the gas for example. Look of course I have a terrible disability, meaning that my short-term memory is unbelievably bad.’* (P6, male, 87 years).

### Theme 3: the influence of an existing care network

Most participants had a large care network including healthcare providers such as the general practitioner (GP), cardiologist or a hospital-based cardiac nurse specialist and informal caregivers such as a partner or children. Participants reported that the community nurse and physical therapist collaborated together and with other involved healthcare providers. Participants remembered that the community nurse consulted the GP, cardiologist or the pharmacist to discuss abnormal vital signs, increased weight or medication-related problems which often resulted in medication changes.

*R2: ‘Those medicines were changed several times ( … ).’*

*I: ‘Was it difficult for you that they were changed so frequently?’*

*R1: No, actually, but I do not know which medicines I should have then, then everything is just all let loose [in multi-dose drug dispenser].*

*I: ‘Okay and the community nurse helped with that, I understand?’*

*R1: ‘Yes, the hospital told her [community nurse] which ones had to get out.’* (P3, male, 81 years)

Participants with an extended healthcare provider network experienced the TCI as an extra appointment within an already busy schedule of care-related appointments:

*‘Once [number of sessions of the physical therapist per week], I think that is enough, yes, I am terribly busy this week. Yesterday I saw the physical therapist, today you are here [interview], tomorrow I have to go to radiology, on Thursday I will see the thrombosis service … The following week, then I have to go back to umm, the surgeon. Yes, I mean you still have so many appointments.’* (P10, female, 82 years).

In addition, some participants preferred the familiar relationships with their own healthcare providers instead of the short-term involvement of the community nurse and physical therapist. For example, one participant already had physical therapy before admission and did not accept additional home-based CR from the TCI. Another chronic heart failure participant had easily accessible contact with the hospital-based heart failure nurse specialist and consulted her instead of the community nurse in case of deviant symptoms:

*‘I must have a systolic pressure of 100 and no higher. ( … ). So, then I contacted A. [cardiac nurse specialist] when my blood pressure was too high. Because of course she obviously knows that too. ( … ) Then it turned out that she had passed a medication to the doctor, and from the doctor it went to the ward, but there had been a hitch when they forgot about this medicine.’* (P6, male, 87 years)

Besides the formal healthcare provider network, most participants also had informal caregivers nearby. Caregivers who were present during the interviews reported that they were involved in the TCI. For example, the partner in the quote below was involved in basic care and administering insulin since hospital discharge as her loved one was too weak post-discharge to take care of himself. She felt supported as she could discuss worries and ask questions to the community nurse about practical care such as the insulin injections:

*‘Yes, I then asked for [advice]. About the quantity of syringes and [umm], but she also told you where is the best place to inject ( … ). She gave some good advice.’* (Partner P8, male, 82 years)

The presence of informal caregivers also had a strengthening effect on participants’ therapy adherence. They often reminded the participant to perform the exercises from the physical therapist on a daily basis:

*‘I think it is incredibly good. I am also always attending so that I see what exercises he has to do. So then at least once a day I call "and now it is for all those exercises".’* (Partner P8, male, 82 years)

In participants with a small or no informal caregiver network, the CCB program was also experienced as additional support:

*‘Well, because I have that big stick behind the door. ( …*) *And my husband thinks so too. He is away a lot. He also works now and then. It helps that there is still a little control [over the medicines].’* (P16, female, 71 years).

## Discussion

This study explored frail older cardiac patients’ experiences with a nurse-coordinated TCI. In general, participants appreciated the care they received within this intervention, and especially felt supported by the home visits in the post-discharge phase. However, participants with severe comorbidities did not always recognized the TCI as a personalized program. Participants with an extended (in) formal caregiver network were satisfied with the TCI, although they preferred to consult their existing network when needed. The results of this qualitative study contribute to an understanding on how the trial participants responded to the intervention and help to interpret the neutral study outcomes on hospital readmission and mortality within 6 months [14]. Three themes emerged from the data: 1) appreciation of care continuity; 2) varying experiences with recovery, and 3) the influence of an existing care network.

Regarding the first theme *appreciation of care continuity,* participants were positive about the delivered care in the clinical, discharge and post-discharge phase although they had some difficulties to distinguish the TCI from usual care. Participants who were able to remember the face-to-face handover of the integrated care plan in the clinical phase were positive about this visit from the community care nurse. Previous research showed that communication (e.g. effective handovers) between care settings contributes to patient satisfaction and is essential to ensure care continuity [27, 28]. Furthermore, participants appreciated the home visits of the community care nurse and physical therapist. Especially, interventions such as the measurement of vital signs, medication management and home-based rehabilitation were mentioned as of great value. Participants felt that the community nurse and physical therapist kept an extra eye on them post-discharge, which contributed to medication adherence and a sense of security to perform CR exercises. Previous studies also reported that patients felt safe when preventive home visits were delivered [29, 30]. However, participants had some difficulties to mention the specific role of the community care nurse which was primary to recognize health deterioration early. Darby et al. [31] previously examined the experiences of geriatric hospitalized patients and also described that patients did not recognize that observing and monitoring their health was part of the actual treatment. Therefore, it is possible that participants mostly experienced that the community nurse visited them without realizing that prevention of health deterioration was the main goal.

Regarding the second theme *varying experiences with recovery,* participants positively valued the home-based CR by the physical therapist and experienced that this has contributed to their functional recovery and self-confidence in their own abilities. This is in line with other studies that examined participants’ experiences regarding rehabilitation [32, 33]. However, some participants with severe comorbidities experienced the physical therapy as too intensive. Although not measured, it is possible that these patients experienced apathy and therefore were less motivated. Apathy is a common geriatric condition around hospital admission [34] and independently associated with an increased risk of functional decline, frailty and cardiovascular disease [35, 36]. These participants had less personalized rehabilitation goals and seemed less motivated for physical therapy. We observed that participants who were able to formulate personal rehabilitation goals were motivated to achieve progress in rehabilitation. Goal setting is essential in rehabilitation as it helps to evaluate the rehabilitation progress and is associated with increased patient motivation and satisfaction with care delivery [37–39]. Therefore, more attention on goal setting and recognition of apathy in frail older cardiac patients may be needed in the education of physical therapists for home-based CR.

We included a severe frail older population which was observed from participant characteristics [14] and the experiences of healthcare providers within this TCI. The mean age was 82.4 years old, 45% had an unplanned hospital admission in the previous 6 months, 31% of patients was cognitively impaired (MMSE 15–23), and geriatric syndromes such as (risk of) delirium (56%), ADL-limitations (39%), falling (47%) and malnutrition (33%) were common. In both groups, 50% of patients reached the composite outcome of unplanned hospital readmission or mortality at 6 months follow-up. In addition, caregivers in our TCI reported that the levels of frailty of the population influenced the performance of the intervention, for example due to comorbidities that impeded patients in physical rehabilitation [24]. This is comparable with the results that we found in the theme *varying experiences with recovery.* We therefore hypothesize that some patients in this TCI were beyond the reach of preventive strategies because of their high age in combination with comorbidities and frailty, and improvement in functional status was no longer feasible. It is important to consider what participants could benefit from home-based CR and for what patients palliative interventions focussing on quality of life [40] would be more suitable.

In theme 2, *varying experiences with recovery, p*articipants further reported that they were unsure if the home visits by the community nurse contributed to their recovery. It was observed during the interviews that participants reported nurses’ interventions (e.g. consultation with the GP about the blood pressure) during the home visits but not recognized their importance to prevent complications. Bleijenberg et al. [41] previously described that older patients appreciated proactive nurse-led home visits when the timing was in line with their needs. It is possible that, after early signalling of health deterioration by the community nurse, proactive interventions were applied before participants noticed that action was needed. This is in line with the experiences of community nurses within this TCI who reported that they contributed to the prevention of complications by early signalling health deteriorations (e.g. heart failure decompensation) [24]. In addition, one of the community nurses experienced that patients thought that they were able to recognize their heart failure deterioration early. However, her experience was that patients overlook the first signals of health deterioration and that early observation and intervening by the community nurse was important to prevent adverse events. This might explain why participants only reported that the community nurse consulted the hospital about the medication while the actual action might have been the prevention of a hospital readmission.

The third theme *the influence of an existing care network* showed that the participants in this TCI mostly had a large formal and informal caregiver network. Participants experienced that the community nurse and physical therapist collaborated with other healthcare providers. Also, the informal caregivers were sufficiently involved in the intervention, for example in education by the community nurse. A protocol for the content of the intervention was used within this TCI which was individualized as much as possible. However, we observed that participants with a large and more familiar healthcare provider network experienced the intervention as intensive and additional to their already busy schedule of care-related appointments. Therefore, the home visits might also be proactively performed by a familiar healthcare providers such as a nurse practitioner working at the general practice. Furthermore, some chronically ill participants seemed to have well self-management skills and were able to easily consult the heart failure nurse specialist themselves in case of a deteriorating health situation. It is known that care coordination across care transitions is important to ensure safe and efficient transitions in care and to reduce the risk of adverse outcomes [3, 7]. As all included patients were at high risk of readmission and mortality [14], also older cardiac patients with an existing care network and participants with self-management skills might contributed from a TCI. However, for future purpose, it is important to identify what patients might benefit most from such interventions. Furthermore, the intervention intensity and content of TCIs could be more personalized to participants’ needs to improve patient satisfaction and efficiency of care.

### Strengths and limitations

We were able to provide important insights into the experiences of older cardiac patients within a nurse-coordinated TCI to better understand the neutral study findings of this trial. As this population is often excluded from clinical trials, their perspectives on participation in research are of added value. The identified themes in this qualitative study contribute to the further development of transitional care interventions for older cardiac patients.

This study also had some limitations. First, this qualitative study was performed within the first 3 months after the intervention was completed. Participants had difficulties to recall their experiences with the TCI, especially in the clinical and discharge phase. Therefore, it was difficult for patients to specifically recall their experiences regarding some key elements of the intervention and to distinguish usual care from care they received within the intervention. We were able to supplement participants’ experiences with data from the logbooks in which involved healthcare providers reported the intervention delivery. This contributed to a more complete view of the intervention delivery in interviewed participants and put the qualitative results in perspective. Second, socially desirable answers could not be fully excluded and may have influenced participants’ answers on their experience with the TCI. Third, selection bias might have occurred as we were unable to examine the experiences of participants whom were deceased soon after inclusion, had withdrawn informed consent in the TCI or did not consent to participate in this qualitative study (*n* = 4). It is possible that their opinions would have resulted in other experiences. Nevertheless, we believe the current selection of patients are representative for the study population in this study.

## Conclusions

The results of this qualitative study contribute to an explanation of the neutral study. For future purpose, it is important to identify which patients might benefit most from TCIs. Furthermore, the intensity and content of TCIs could be more personalized by tailoring interventions to older cardiac patients’ needs, considering their frailty, self-management skills and existing formal and informal caregiver networks.

## Supplementary Information

**Additional file 1.** Interview guide.

## Data Availability

The datasets used and/or analysed during the current study are available from the corresponding author on reasonable request.

## Abbreviations

CGA: Comprehensive geriatric assessment
CN: Community nurse
CR: Cardiac rehabilitation
I: Interviewer
IQR: Interquartile range
PT: Physical therapist
R: Respondent
RCT: Randomized clinical trial
SD: Standard deviation
TCI: Transitional care interventions
