# Supplementary material for: Experiences of frail older cardiac patients with a nurse-coordinated transitional care intervention - a qualitative study
Source: BMC Health Serv Res. 2021 Aug 10;21:786. doi: 10.1186/s12913-021-06719-3 (PMC8353821; doi:10.1186/s12913-021-06719-3)
Supplement: Supplementary file 1 — Additional file 1. Interview guide. [file 12913_2021_6719_MOESM1_ESM.pdf]

Experiences of frail older cardiac patients with a nurse-coordinated transitional care intervention - a qualitative study

Patricia Jepma[1,2*], Corine H.M. Latour[2], Iris H.J. ten Barge[3], Lotte Verweij[1,2], Ron J.G. Peters[1], Wilma J.M. Scholte op Reimer[1,2], Bianca M. Buurman[1,4]

[1] Department of Cardiology, Amsterdam UMC, University of Amsterdam, Amsterdam, the Netherlands.
[2] Center of Expertise Urban Vitality, Faculty of Health, Amsterdam University of Applied Sciences, Amsterdam, the Netherlands.
[3] Nursing Sciences, program of Clinical Health Sciences, University Medical Center Utrecht, Utrecht, The Netherlands.
[4] Department of Internal Medicine, Section of Geriatric Medicine, Amsterdam UMC, University of Amsterdam, Amsterdam, the Netherlands.

Corresponding author:

Patricia Jepma
Amsterdam University Medical Centre, department of Cardiology
Centre of Expertise Urban Vitality, Amsterdam University of Applied Sciences
Tafelbergweg 51 Amsterdam
1105 BD the Netherlands
E-mail: p.jepma@amsterdamumc.nl
Phone: +31650440499

Supplemental Digital Content 1: Interview guide

This interview will be about your experiences with the care you received after being admitted to the [name of hospital] at [name of ward] because of problems with your heart.

Opening question:

1. I understand that you were admitted for (designate the diagnosis)? Is that true?
   a. How are you now?

Clincal phase:

While at the ward in the hospital, you started participating in a scientific study referred to as the XXX study. To this end, you signed an informed consent form and a study nurse asked you a number of questions about the condition of your health prior to and during hospital admission. Can you still remember this? {If yes, continue to question 2}

No? It was a rather long questionnaire that was administered to you by a study nurse who completed the answers on a small computer screen that she had with her. For example, you were asked questions about your daily functioning (whether you could still wash yourself, change your clothes, runs errands) and about fatigue, your fear of falling, your appetite, the medicines you are taking and whether you are satisfied with your life. Do you still remember this? {Yes, go further to question 2} {No, skip to question 3}

2. Do you know why this questionnaire was administered to you?
   a. Yes? Can you explain that?
3. Did you get the feeling that you could also tell your own story during this interview?
   a. Can you tell us a bit more about that?
4. How stressful did you find this questionnaire?

In addition to the questionnaire, you also had to do some physical exercises, such as… Can you still remember that? {Yes, go further to question 5} {No, skip to question 7}

5. Do you know why these physical tests were done on you?
   a. Yes? Can you explain that?
   b. No?
6. How stressful did you find these physical tests?

Using the questionnaire and some physical tests, the study nurse assessed which health symptoms needed further attention during and after hospitalisation and a treatment plan was accordingly drawn up.

7. Have you discussed the results of the questionnaires and tests?
   a. Yes? Do you remember what was discussed with you?
      i. Can you say what you thought about that?
   b. No? Did you want the results of the tests to be discussed with you?

Using the treatment plan with goals, we wanted to see if we could facilitate your recovery.

8. Were there things you wanted to achieve yourself when you got back home?
   a. Yes? Can you explain that?
   b. Were these goals taken into consideration when drawing up the integrated care plan?
      i. Yes? Can you tell us a bit more about that?

ii. No? Can you indicate exactly what you missed or how you would have wanted it differently?

Discharge phase

9. At one point you were almost allowed to go home. Can you tell us how the preparations for the discharge went and how you experienced this?
   a. Were there any other things that needed to be arranged for you before you went home?
      i. Yes? Can you tell us about everything that had to be arranged?
   b. Did you feel involved in everything that needed to be arranged for discharge and were you consulted?
      i. Yes? Can you tell us a bit more about that?
      ii. No? Can you indicate how you would have wanted to be more involved?

10. Did the community nurse visit you in the hospital?
   a. Yes? How did you feel about the community nurse visiting you in the hospital before? (Deepening; what exactly was nice or not?)
      i. Can you tell us what you thought of the conversation with the community nurse in the hospital?
      ii. Were there other things you would have wanted to discuss during this visit from the community nurse in the hospital?
   b. No? How would you have felt if you had already met the community nurse in the hospital?
      i. Yes? Can you explain that?

11. Can you tell me if you were confident to go home, were you ready for discharge?
   a. Yes? Can you tell us what you had confidence in?
   b. No? Can you tell us what you dreaded?

Post-clinical phase:

I now want to talk to you about the period back at home:

12. Can you tell us how you experienced the first period at home?
   a. Did you still need care at that time?
      i. Yes, what care did you need and who helped you with that?
   b. Can you tell us whether you were confident that this care was properly arranged?
   c. Can you tell us what was most difficult for you when you returned home?
      i. Did you discuss that with someone? (for example, the community nurse? With family or relatives? Or other caregivers?)
   d. Can you also tell us what was not so bad for you when you returned home?

We know that people are sometimes uncertain about their health status after being admitted to hospital. Such as symptoms that you may still have because of the hospitalisation, your condition, or things that you were able to do independently before admission but that are now more difficult.

13. Do you recognise that you have ever been unsure about something after being discharged from hospital?
    a. Yes? Can you tell us a bit more about that?
    b. Have you ever discussed these uncertainties with someone?

14. Did you have any other symptoms (physical or otherwise) when you were at home?
    a. Yes? Can you tell us about this?
    b. Have you ever discussed these symptoms with someone? (for example, the community nurse? With family or relatives? Or other caregivers?)
        i. Yes? With whom and how could they support you?
        ii. No? Why not?

15. Were there any family or relatives who were involved in the support when you returned home?
    a. Yes? Can you tell us about the role they play in the support?
    b. How do you experience the involvement of your family or relatives?

Community nurse

As I mentioned to you earlier, a community nurse [name] should have visited you during the first few weeks after discharge and she came to discuss how you were doing after your hospitalisation. Is that correct and do you remember it? {Yes? Go to question 16} {No? Go to question 17}?

16. Can you tell us a bit about the visits of the community nurse; and what you were talking about?

17. The community nurse visits patients to support their recovery after hospitalisation, for example by looking at your medicines with you, or discussing your fluid and diet intake and often your blood pressure is also monitored.
    a. Did she talk to you about the medicines, for example?
        i. Yes? Could you tell us more about what she then discussed with you?
        ii. No? What do you think about that? Would you have wanted the community nurse to have discussed this with you?
    b. Did you talk about, for example, your lifestyle (healthy food, exercise in daily life)?
        i. Yes? Could you tell us more about what she then discussed with you?
        ii. No? What do you think about that? Would you have wanted the community nurse to have discussed this with you?
    c. (If possible) You just said that you sometimes had… complaints at home. Is that also something that you have discussed with the community nurse?
        i. If so, what did she do about your complaints?
        j. If not, do you know why you did not discuss this with her?

18. Did you feel that the community nurse took your wishes into consideration?
    a. Yes? Can you tell us a bit more about that?

b. No? Can you tell us whether and how he/she could have had more due consideration to your wishes?
c. To what extent did the community nurse encourage you to remain independent?

19. Did you trust the expertise of the community nurse?
    a. Can you say why or why not?

20. Do you know approximately how frequently the community nurse has visited your home?
    a. Was the number of visits sufficient for you, or did you think the number of visits was too many or too few?
    b. Was the number of visits spread nicely over the time for you? Was there too much or too little time in between?

Physical therapist

In the first few weeks, a physical therapist [name] had also visited you a number of times. Can you still remember that? *{No? The physical therapist has probably worked with you on your condition and given you exercises to perform on your own, such as getting up from a chair several times, and perhaps doing squats, is that correct?}* {No: go to question 22, Yes: go to question 17}

21. Can you tell us about the visits of the physical therapist to your home, what exactly did he/she do?

The physical therapist sometimes helps people set goals to return to activities that may have been made more difficult by the hospital admission. Sometimes the physical therapist exercises together to facilitate doing this independently again.

22. Was it jointly discussed with you what you would like to achieve with the visits of the physical therapist?
    a. Yes? Can you tell us a bit more about that?
    b. No? Can you tell us how he/she could have had more due consideration to your wishes here?

23. Did the physical therapist give you exercises that you could perform yourself when the physical therapist was not present?
    a. No? Do you know why you did not get any exercises?
        i. What do you think about that?
    b. Yes? What did you think of the exercises?
    c. Were the exercises too difficult, exactly right or too easy?
    d. Were you successful in performing these exercises even when the physical therapist was not there?
    e. Did the physical therapist encourage you to get started with the exercises yourself?
        i. Yes? Can you tell us how he/she did that?
        ii. No? How could the physical therapist have stimulated you more?
    f. Were you confident that these exercises also contributed to your recovery?
        i. Can you say why or why not?
    g. Have you ever found it exciting to exercise, even when the physical therapist was with you?

i. Can you say why/why not?

24. Did you trust the expertise of the physical therapist?
    a. Can you say why or why not?

25. Do you know approximately how frequently the physical therapist has visited your home?
    a. Was the number of visits sufficient for you, or did you think the number of visits was too many or too few?
    b. How many times a week did the physical therapist visit you? Was the number of visits spread nicely over the time for you? Was there too much or too little time in between?

26. Did the community nurse and the physical therapist also visit you home together/at the same time?
    a. Yes? Can you tell us how this visit came about?
    b. Do you also know why they visited your home together?
        i. What has this visit contributed to for you?
        ii. How were you involved in this joint visit?

27. Did the community nurse or physical therapist ever have contact with the hospital or your general practitioner?
        i. Yes? Do you know what this was for?
        ii. How did you feel about them doing that and then involving you with it?

28. I understand that many caregivers visited you. What do you think about that?

Closure:

29. Are you satisfied with the current condition of your health and what you have achieved since your discharge from the hospital?
    a. Yes? Do you feel that the support of the community nurse has contributed to this? And the physical therapist?
        i. Which support has contributed something for you and which support has not?
    b. No? Can you indicate why?

30. Have you been readmitted to hospital recently?
    a. Yes? What was the reason for this readmission?
    b. How did that go?
    c. Were any more caregivers involved in the readmission?
        i. Yes? Which caregivers were involved and how?
        (Emphasis on the community nurse and physical therapist at home)

(Only ask this question if appropriate)
I hear you say that xxx went wrong, but is it true that this gives you the impression that the readmission was unnecessary?

31. Could the admission have been prevented in your opinion?

i. If so, how do you think it could have been prevented?

32. Are there any aspects you missed in this interview, which you think are important for me to know?

May I thank you very much for this interview. You have told me a lot in a short time, we will deal with the information xxx as follows and we will use it xxx.
